# The epitranscriptomic m6A RNA modification modulates the synapse in ageing and in a mouse model of synucleinopathy

**DOI:** 10.1038/s41531-026-01362-3

**Published:** 2026-05-06

**Authors:** Avika Chopra, Mary Xylaki, Fanzheng Yin, Ricardo Castro-Hernández, Madiha Merghani, Valentina Grande, Brit Mollenhauer, André Fischer, Tiago F. Outeiro

**Affiliations:** 1 Department of Experimental Neurodegeneration, Center for Biostructural Imaging of Neurodegeneration, University Medical Center Göttingen, Göttingen, Germany; 2 Department of Neurology, University Medical Centre Göttingen, Göttingen, Germany; 3 Department for Systems Medicine and Epigenetics, German Center for Neurodegenerative Diseases (DZNE), Göttingen, Germany; 4 Paracelsus Elena Klinik, Kassel, Germany; 5 Department of Psychiatry and Psychotherapy, University Medical Center Göttingen, Göttingen, Germany; 6 Cluster of Excellence “Multiscale Bioimaging: from Molecular Machines to Networks of Excitable Cells” (MBExC), University of Göttingen, Göttingen, Germany; 7 Faculdade de Medicina e Ciências Biomédicas, Algarve Biomedical Center Research Institute, Universidade do Algarve, Faro, Portugal; 8 Translational and Clinical Research Institute, Faculty of Medical Sciences, Newcastle University, Newcastle Upon Tyne, UK; 9 Scientific employee with an honorary contract at Deutsches Zentrum für Neurodegenerative Erkrankungen (DZNE), Göttingen, Germany

**Keywords:** Molecular biology, Neuroscience

## Abstract

N^6^-methyladenosine (m6A) is the most abundant transcriptional modification in eukaryotic RNA, regulating RNA fate. While the functions of m6A in the development of the mammalian brain have been extensively studied, its role in synaptic plasticity, and brain function remain underexplored. The involvement of this modification in Parkinson’s disease and other synucleinopathies has only recently been studied, and needs further investigation. Here, we investigated the m6A epitranscriptome using MeRIP-seq in A30P-aSyn transgenic mice (aSyn Tg). We observed hypermethylation of synaptic genes in young aSyn Tg mice compared to age-matched control mice. The methylation was reduced during ageing. Using immunofluorescence imaging and biochemical analysis, we further investigated the levels and distribution of m6A regulatory enzymes—writer, METTL3, reader, YTHDF1, and eraser, FTO, in the cortex, striatum, hippocampus, and cerebellum of aSyn Tg and control mice, and in primary cortical neuronal cultures. While the levels of these proteins were similar, METTL3 was found in the nucleus and in the post-synaptic compartment in neurons, suggesting it may play a role in methylation at the post-synapse. Our findings suggest that alterations in the regulation of m6A RNA methylation may be associated with neurodegeneration and ageing and it may play a significant role at the synapse.

## Introduction

Epigenetics is a process that acts as a bridge between genotype and phenotype, changing the outcome of a gene without modifying the DNA sequence. It encompasses various types of alterations such as DNA methylation, histone modifications, and chromatin remodelling^1,2^. The field that studies posttranscriptional changes of RNA bases is known as epitranscriptomics and it aims to identify modifications in the transcriptome and unravel their regulatory roles^3^. N^6^-methyladenine (m6A) modification is the most prevalent mRNA modification in eukaryotes, occurring at a frequency of 0.15–0.6% of all adenosines^4–6^ and is found in different types of RNAs including protein coding, messenger RNA (mRNA)^7,8^. This modification is being extensively studied due to its prevalence and importance in the regulation of various cellular functions including mRNA stability, splicing, export, decay, and translation^8–13^. These methylation marks are mostly added co-transcriptionally to specific consensus motif sequences and are mostly found close to the 3’ un-translated region (3’-UTR) of the mRNA, at the stop codon^4,14^.

The m6A modification machinery consists of three groups of enzymes, namely methyltransferases - writers, demethylases – erasers, and detectors of this modification - readers. The modification can be added onto the RNA by methyltransferases including methyltransferase-like 3 (METTL3), methyltransferase-like 14 (METTL14), and adaptor protein Wilms tumour 1-associated protein (WTAP)^5,15–20^. METTL3 was the first methyltransferase identified, and was described as a key component in the methylation of transcripts involved in physiological processes such as embryonic and brain development^21–24^. The catalytically active part of METTL3 forms a heterodimer with METTL14, which provides the structure to install the m6A modification onto RNA^5,25–28^. Apart from these, the m6A methyltransferase complex comprises other proteins, including RNA binding proteins (RBP) like RBP15, which recruit this machinery to the target mRNA to facilitate methylation^16,29^. Demethylases, for instance, fat mass and obesity-associated protein (FTO) and alkB homologue 5 (ALKBH5) remove the m6A modification marks, making methylation a reversible and dynamic process^30,31^. FTO was the first eraser protein identified^30,32^ and it carries out oxidative demethylation, where m6A is converted in several steps to N^6^-hydroxymethyl adenosine (hm6A), then to N^6^-formyladenosine (f6A) and lastly to adenine^32^. Additionally, reader proteins like the YT521-B homology (YTH) domain family of proteins including YTHDF1, YTHDF2/3, insulin-like growth factor 2 mRNA-binding proteins (IGF2BP1), heterogeneous nuclear ribonucleoprotein (HNRNPA2B1) recognise the modification and modulate alternative splicing and processing of target transcripts^8,13^. YTHDF1, YTHDF2, and YTHDF3 are located in the cytoplasm. The main function of these proteins is unclear; however, previous studies have reported different roles of these enzymes in a cell. While YTHDF1 promotes the translation of mRNA, thereby promoting its protein expression, YTHDF2 promotes the degradation of mRNA, and YTHDF3 promotes both translation and degradation of mRNA^33,34^.

While m6A methylation is involved in important cellular functions, such as regulating cell growth, development, and differentiation, it has been associated with neurodegeneration and ageing. During ageing, a significant increase in the levels of m6A have been reported when 2-weeks old mice were compared with mice older than 6-weeks^35^. In a study conducted in humans, it was observed that the levels of m6A methylation were elevated in patients with intervertebral disc degeneration, which is only observed in ageing^36^. Another recent study highlighted the relationship between elevated m6A levels and brain ageing in both mice and humans^35^, although a significant downregulation of m6A methylation has also been reported in ageing mice and in postmortem brain tissue from Alzheimer’s disease patients^37^. Importantly, ageing is considered a major risk factor for PD as it affects multiple homoeostatic pathways including mitochondrial function and protein degradation pathways, ultimately leading to cell death in the substantia nigra^38–40^.

Neurodegenerative disorders such as PD and related synucleinopathies, are associated with the accumulation of aggregated alpha-synuclein (aSyn) in the brain, ultimately associating with neuronal stress and cell death. Despite the growing interest in the role of m6A methylation in ageing, its role in PD is still unclear and remains controversial. While a recent study indicated that the expression levels of m6A are significantly reduced in cellular models of PD and the striatum of a rodent model of PD^41^, a different study showed that the m6A methyl transferases were significantly decreased and demethylases were increased in the substantia nigra and the striatum of mouse models of PD^42^. METTL3 knock-out mice show reduced m6A levels and shortened lifespan, alongside degenerative alterations in bone, suggesting that m6A methylation is crucial for a healthy ageing^43^. An in-vitro model of PD, where neurons were treated with 1-methyl-4-phenylpyridinium (MPP + ), revealed a reduction in the levels of METTL14, in turn triggering neuroinflammation and pyroptosis^44^.

Here, we investigated the m6A RNA modification profile in the context of ageing and neurodegeneration in a mouse model of synucleinopathy, using the A30P aSyn transgenic (aSyn Tg) mouse model. A30P aSyn Tg mice develop motor symptoms and the accumulation of aSyn in the spinal cord after 12 months of age^45^. We performed methylated RNA immunoprecipitation sequencing (MeRIP-seq) to obtain the m6A epitranscriptome of the midbrain of C57BL6 (Wt) control and aSyn Tg mice at 3 months (3 mo) and 15 months (15 mo) of age. We observed an increase in the m6A methylation in the genes involved in the molecular and biological processes at the synapse. Several genes involved in synaptic processes were hypermethylated in 3 mo aSyn Tg vs. Wt mice, while the methylation marks were reduced in 15 mo aSyn Tg vs. age-matched Wt mice. Using immunofluorescence imaging of primary cortical neurons, we assessed the presence and levels of proteins METTL3, YTHDF1, and FTO which have been studied in the context of PD and in other neurodegenerative disorders^46–48^. We observed that the localisation of the writer enzyme, METTL3 is not only limited to the nucleus, but is also present at the post-synapse. Our findings suggest that m6A RNA methylation may play a role in the development or progression of neurodegeneration and ageing, and may be involved in regulating synaptic function.

## Results

### m6A alterations during ageing in Wt mice and in a transgenic model of synucleinopathy

**Fig. 1:**
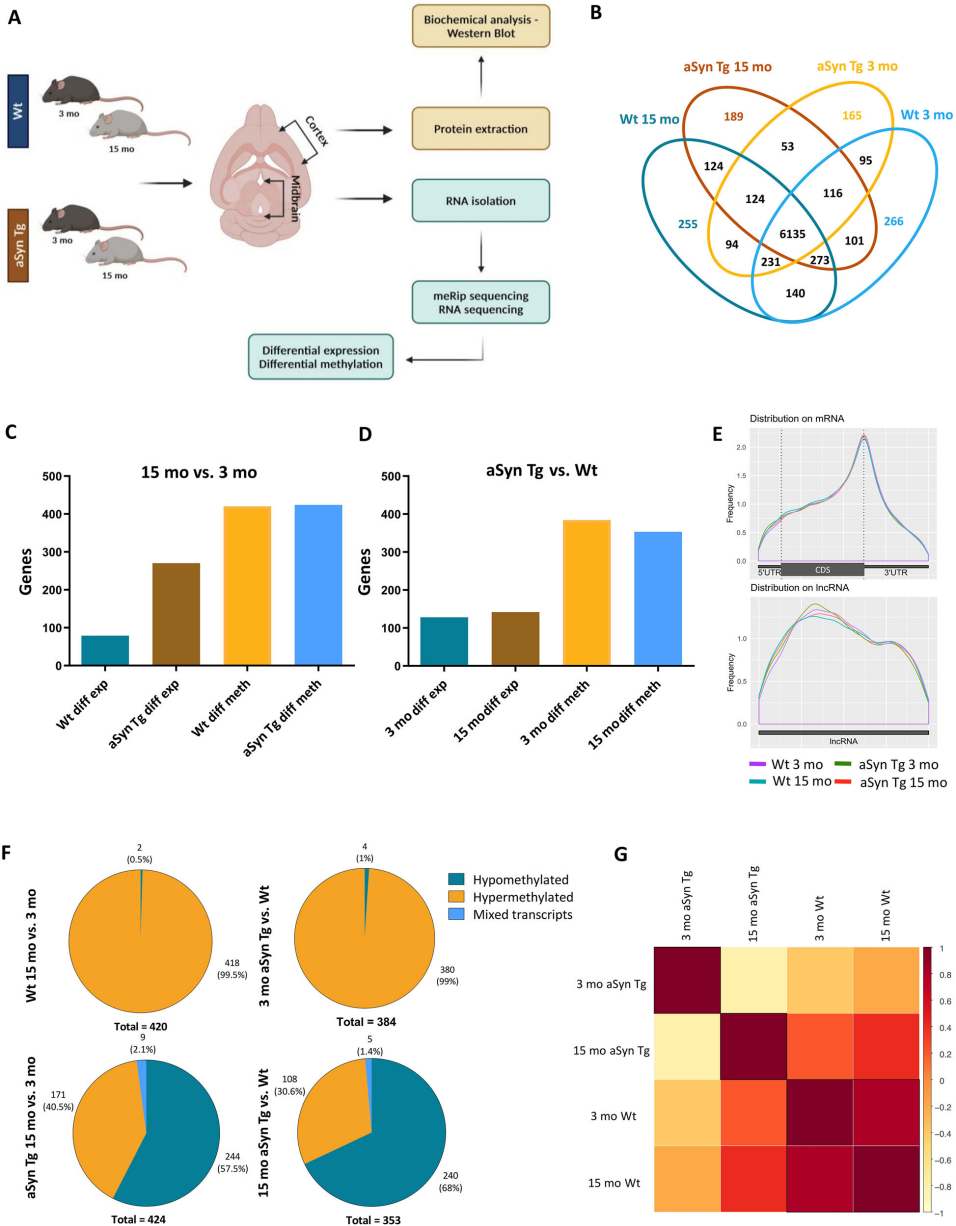
m6A modification landscape in ageing and aSyn Tg mice. **A** Overview of protocols utilised in this project. Five mice of each Wt and aSyn Tg were used for RNA sequencing analysis. **B** Venn diagram representing the number of differentially methylated genes involved in young and old Wt and aSyn Tg mice. It also provides a summary of the overlapping genes across different genotypes. **C** Bar plots representing the variations in the differentially expressed genes and differentially methylated genes in the midbrain from aSyn Tg mice than Wt mice when 3 mo mice are compared with 15 mo applying equal cutoffs for FC and adjusted p-value (FC > 1.2 and p-adj ≤ 0.05). Left: with age, right: with aSyn (**D**) Bar plots representing the effects of aSyn pathology on differential expression and differential methylation in 3 mo and 15 mo Wt vs. aSyn Tg mice. **E** Guitar plots representing the distribution of m6A methylation on mRNA and lncRNA. CDS stands for coding sequence, and UTR stands for untranslated region. **F** Pie charts representing the overview of the distribution of hypomethylated, hypermethylated and mixed transcripts as an effect of aSyn pathology and ageing. **G** Heatmap displaying the Spearman correlation between differentially methylated transcripts in 3 mo and 15 mo Wt and aSyn Tg mice.

Firstly, we began by examining the distribution of differentially expressed transcripts and transcripts with m6A methylation in the healthy ageing brain of Wt mice. We further extended the analysis to aSyn Tg mice, to compare the effects of ageing on m6A modification and assess this change in the context of synucleinopathy (Fig. 1A). We extracted and dissected the midbrains from five 3 mo and five 15 mo Wt and an equal number of aSyn Tg mice, as this brain region is particularly relevant in the context of PD and related synucleinopathies. There was no correlation identified between m6A methylation marks and differential expression of transcripts (Supplementary Fig. S4). We found a total of 6135 methylated transcripts in common between all groups (Fig. 1B). The 3 mo Wt mice displayed 266 unique methylated transcripts and 255 were identified in the 15 mo Wt mice. These numbers were lower for the aSyn Tg group, where the 3 mo mice showed 165 unique methylated transcripts and the 15 mo mice showed 189. At 15 months, both genotypes showed 124 commonly methylated transcripts, suggesting the occurrence of conserved processes in normal ageing and synucleinopathies. The aSyn Tg mice displayed 53 methylated transcripts correlated with aSyn expression and conserved with ageing.

Next, we compared the differential expression of genes due to ageing in 15 mo vs 3 mo mice in both Wt and aSyn Tg animals. We observed a stronger effect in aSyn Tg mice where 270 transcripts were differentially expressed when compared with Wt mice, where only 79 transcripts were differentially expressed (Fig. 1C). Most differentially expressed transcripts in aSyn Tg mice were downregulated (Supplementary Fig. S1C). Additionally, methylated RNA immunoprecipitation sequencing (MeRIP-seq) was performed to determine the midbrain-specific epitranscriptomic landscape in 3 mo and 15 mo mice. However, when we compared the differentially methylated transcripts, we found similar numbers between Wt (420 genes) and aSyn Tg (424 genes) mice (Fig. 1C).

On the other hand, the effect of aSyn-A30P overexpression was assessed by a comparison between aSyn Tg and Wt mice at 3 mo and 15 mo. We did not identify major differences in the number of differentially expressed transcripts which were 128 at 3 mo and 142 at 15 mo. Similarly, the differentially methylated transcripts showed no significant changes between Wt and aSyn Tg at 3 mo (384 genes) and 15 mo (353 genes) (Fig. 1D). While we did not see a significant difference in the differential methylation, we found that the prevalence of hyper- and hypomethylated transcripts was reversed.

The overall distribution of total m6A methylation peaks on mRNA did not reveal any striking anomalies in any of the groups. As expected, the majority of the m6A modifications were localised close to the 3’ UTR on mRNA (Fig. 1E), as previously reported for ageing-related genes^35^.

As a consequence of ageing, the methylation levels of the differentially methylated m6A transcripts in 15 mo vs. 3 mo Wt mice were 418 hypermethylated and only 2 hypomethylated transcripts. On the contrary, 171 genes were hypermethylated and 244 transcripts were hypomethylated in 15 mo vs. 3 mo aSyn Tg mice (Fig. 1F, left panel). A transcript can carry multiple m6A methylation marks and some of these marks likely increase while others decrease simultaneously. Such transcripts are referred to as mixed transcripts. We found only 9 such mixed transcripts (Fig. 1F, left panel) in aSyn Tg mice during ageing.

To identify the effect of aSyn expression on the levels of the m6A modification, we compared 3 mo aSyn Tg mice with age-matched Wt mice. We found 380 hypermethylated transcripts and only 4 hypomethylated transcripts, and no mixed transcripts. On the other hand, when comparing 15 mo aSyn Tg vs. Wt mice, a reverse trend was observed, with 108 hypermethylated, 240 hypomethylated, and only 5 mixed transcripts (Fig. 1F, right panel). The heat map showed a strong correlation when 15 mo Wt were compared with 3 mo Wt methylated transcripts while, on the other hand, 15 mo vs. 3 mo aSyn Tg showed an inverse correlation (Fig. 1G).

### m6A levels are reduced in synaptic transcripts upon ageing

**Fig. 2:**
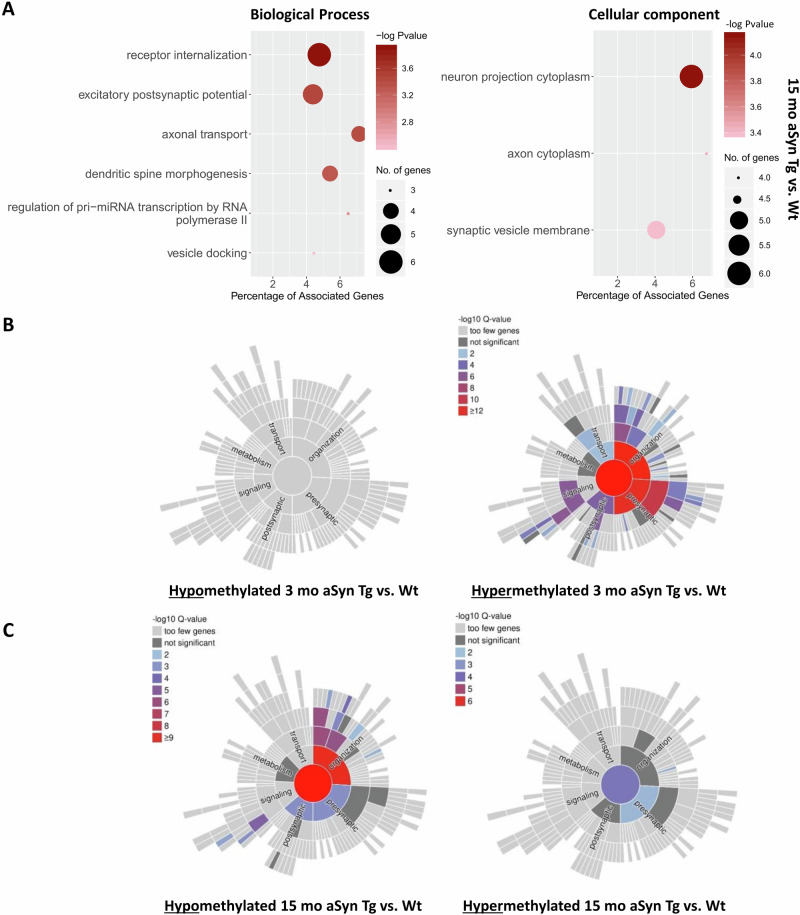
Gene ontology analysis for 3 mo and 15 mo Wt vs. aSyn Tg mice. **A** GO analysis for the biological processes and cellular processes in 15 mo aSyn Tg vs. Wt mice, FDR = 1.5. **B** Sunburst plots representing GO analysis for the genes involved in the processes at the synapse in aSyn Tg vs. Wt mice at 3 mo. Graphs created with syngoportal.org. **C** Sunburst plots representing GO analysis for the genes involved in the processes at the synapse in aSyn Tg vs. Wt mice aged mice at 15 mo. Graphs created with syngoportal.org.

To investigate the cellular processes and the subcellular regions where the differentially methylated transcripts were identified in 15 mo aSyn Tg vs. Wt mice, we performed gene ontology (GO) term analysis. Synaptic function was the biological process most represented in the GO analysis. Our analysis revealed that transcripts with m6A methylation marks have a strong enrichment for biological processes such as receptor internalisation, excitatory post-synaptic potential, axonal transport, and dendritic spine morphogenesis. The identified transcripts were also involved in cellular processes including neuron projection cytoplasm and synaptic vesicle membrane (Fig. 2A). Previous data from our group has also shown that, while the average dendritic spine number was not significantly different, a strong difference was observed in the abundance of different types of spines in aSyn Tg neurons when compared with Wt neurons^49^. A significant reduction was observed in the number of mushroom and thin spines. However, the number of stubby spines was significantly higher in the aSyn Tg mouse midbrain tissue^49^. The increase in stubby spines suggests an increased likelihood of spines potentially developing into mature spines. This aligns with our results where we saw a significantly high number of synapses in aSyn Tg neurons when compared with Wt.

We also observed that when we compared the differentially enriched genes in aSyn Tg 15 mo vs. 3 mo, we saw a strong overexpression of genes involved in pathways related to the immune system (Supplementary Fig. S2). The genes with the highest enrichment score were involved in immune responses including the innate immune system, adaptive immune system, immunoglobulin binding, phagocytic vesicle, defence response, innate immune response, and regulation of immune system processes among others (Supplementary Fig. S2).

We used the SynGO software^50^ (https://syngoportal.org/), an experimentally annotated database for synaptic location and functional GO, to specifically check the m6A methylation of synaptic transcripts. These differentially methylated genes are primarily involved in synaptic organisation, metabolism, transport, signalling, and both pre- and post-synaptic processes. Based on MeRIP and RNA sequencing analysis, sunburst plots of Wt and aSyn Tg mice were generated (Fig. 2B). Our findings suggest that the differentially methylated transcripts that are involved at the synapse and in synaptic function were majorly hypermethylated in 3 mo aSyn Tg mice compared to 3 mo Wt mice (Fig. 2B). These hypermethylated transcripts were primarily involved in synapse organisation and at the pre-synapse (Fig. 2B). Conversely, at 15 months of age, the majority of the differentially methylated transcripts were hypomethylated when aSyn Tg mice are compared with Wt mice, which were largely involved in signalling, pre- and post-synapse, and synapse organisation (Fig. 2C).

In summary, we observed that the differentially methylated genes in 15 mo aSyn Tg vs. Wt mice were mostly hypomethylated and involved in synaptic processes, especially at the pre-and post-synapse. However, at 3 mo, most of the differentially methylated genes were hypermethylated.

### The levels of m6A regulatory proteins do not change in different brain regions in Wt and aSyn Tg mice

**Fig. 3:**
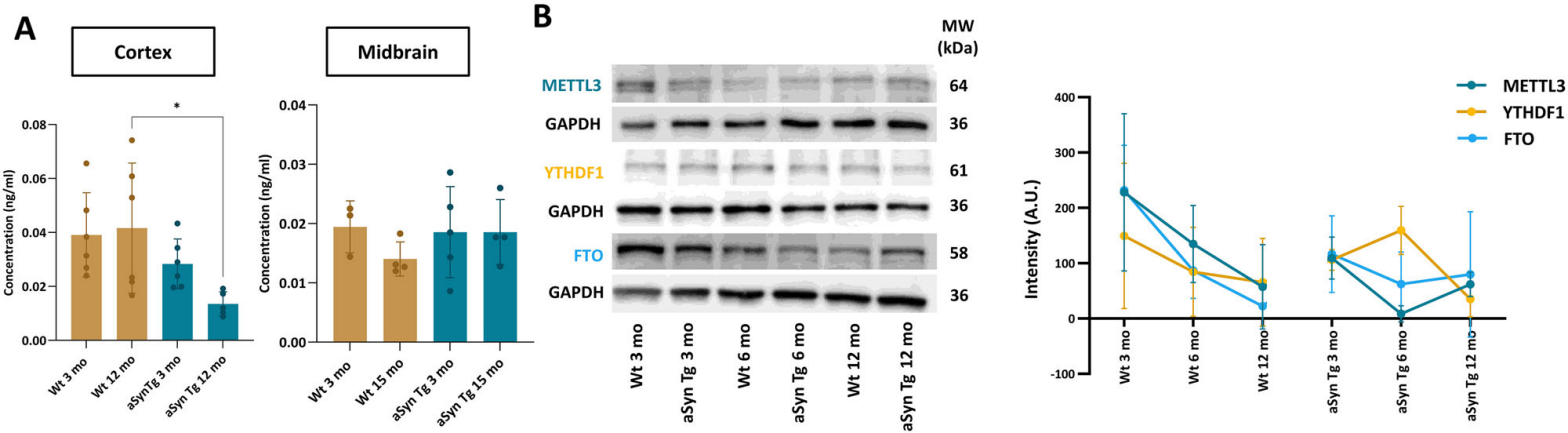
Levels of m6A RNA and m6A regulators in mouse brain tissue. **A** Bar plots representing the levels of m6A modification on total RNA using ELISA from cortical and midbrain regions of Wt and aSyn Tg mice. A significant reduction in the levels of m6A was observed in the aged, 12 mo aSyn Tg mouse cortex when compared with 12 mo Wt mice (*, p-ajd. value = 0.045). No significant differences were observed in the midbrain region of the brain. **B** Biochemical analysis using western blot comparing the expression levels of METTL3, YTHDF1 and FTO in the cortical tissue from 3 mo, 6 mo and 12 mo Wt vs. aSyn Tg mice. Scatter plot showing no significant difference in the expression level when comparing Wt with aSyn Tg mice.

To validate the meRIP-Seq results and to evaluate if the overall levels of the m6A modification are affected in aSyn Tg vs. Wt mice, we performed an m6A ELISA, for total RNA isolated from cortical and midbrain tissue of young and aged, Wt and aSyn Tg mice. We found that overall, the levels of m6A modification marks were higher in the cortex compared to the midbrain. In cortical brain tissue, we observed that the m6A marks in the aSyn Tg mice do not display the same pattern as in Wt animals (Fig. 3A). A significant reduction in m6A marks was observed in 12 mo aSyn Tg mice compared to 12 mo Wt mice (Wt; mean = 0.032 ± 0.018 OD, aSyn Tg; mean = 0.011 ± 0.002 OD, p-adj. value = 0.0364). In younger animals, while not significant, a slight reduction in m6A marks was observed in 3 mo aSyn Tg when compared with 3 mo Wt mice, indicative of an effect that probably starts at a young age and progresses with ageing (Fig. 3A). In the midbrain, we did not observe significant differences in the m6A marks between the groups (Fig. 3A). This is consistent with the MeRIP-Seq data where we observed that the total number of differentially methylated transcripts was not altered among the groups (Fig. 1C, D). This suggests that despite the observed hypo- and hyper-methylation in certain transcripts (Fig. 1F) the overall m6A levels remain in an equilibrium in the midbrain.

Next, we asked if the observed changes in the m6A post-transcriptional marks in the cortex correlate with altered levels of the three m6A regulatory proteins in the cortical brain tissue. To do so, we performed immunoblotting analysis to assess the expression of m6A regulators—METTL3, YTHDF1, and FTO (Supplementary Fig. S3). We used three different age groups–3 mo, 6 mo and 12 mo from the Wt and aSyn Tg mice for our analysis. We observed no significant differences in the expression levels of these m6A regulatory proteins neither within genotypes (aSyn Tg vs. Wt) nor across the different age groups (Fig. 3B). Nonetheless, we observed a reducing trend in the levels of METTL3 and FTO in Wt mice during ageing (Fig. 3B). We observed a declining trend in the levels of METTL3 from Wt 3 mo to Wt 12 mo (p-value = 0.6542). Similarly, for FTO a reducing trend was observed from Wt 3 mo to Wt 12 mo (p-value = 0.1906).

Considering we detected no difference in the protein levels of m6A regulators, we sought to identify if the sub-regional and/or subcellular localisation of the three m6A regulatory proteins is altered. For this, we assessed the levels of METTL3, YTHDF1 and FTO by immunofluorescence intensity analysis in four different regions of the mouse brain, namely cortex, striatum, hippocampus, and cerebellum. MAP2 co-staining was used only to identify the neurons present in the areas of the brain. All these proteins were present in our selected areas of interest, but their compartmentalisation varied across different regions of the neurons.

**Fig. 4:**
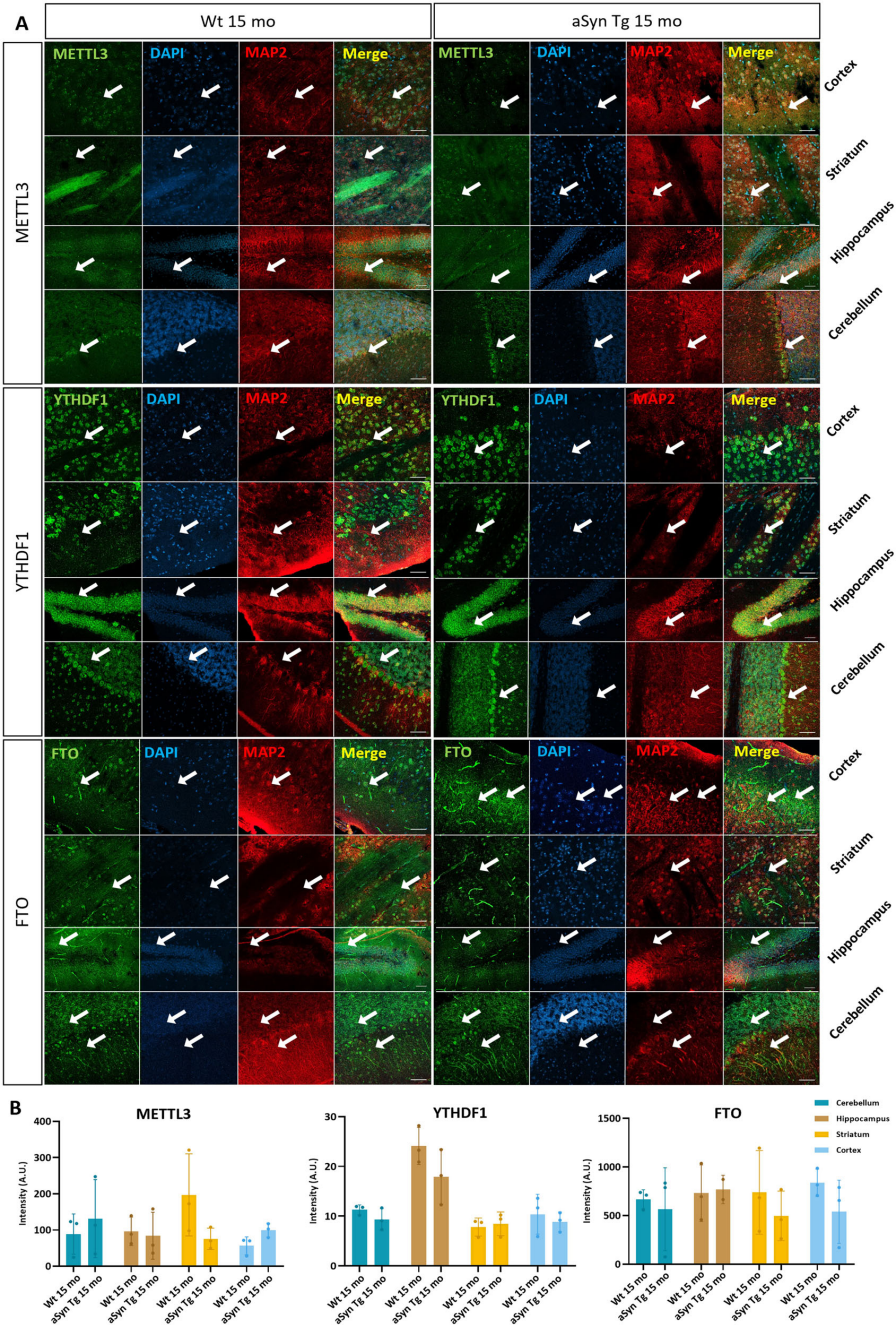
Distribution of m6A regulatory proteins METTL3, YTHDF1 and FTO in the cortex, striatum, hippocampus, and cerebellum of mouse brain. **A** Representative images of mouse brain tissue slices (30 µm) fixed, and stained with MAP2, METTL3, YTHDF1 and FTO. METTL3 was observed both in the nucleus and at the post synapse, while YTHDF1 was present in the soma, and FTO was present in the nucleus and in some dendrites, typically in the cerebellum. The images are acquired using a confocal microscope, 40x magnification with tiles, and the scale bar = 50 µm. White arrows point to the neurons with the expression of the respective proteins. **B** Bar plots showing the comparison between the mean intensity of METTL3, YTHDF1 and FTO in 15 mo Wt vs. aSyn Tg mice. No significant difference as observed between aged Wt and aSyn Tg mice when the levels of m6A proteins were compared. For each marker, three Wt 15 mo and three aSyn Tg 15 mo were included.

The m6A writer protein, METTL3 was observed as small puncta within the nucleus in the cortex, striatum, hippocampus, and cerebellum in both aged Wt and aSyn Tg brain (Fig. 4A). The m6A reader protein, YTHDF1, on the other hand, localised only in the soma of neurons in all four areas of interest, with a similar expression pattern in Wt and aSyn Tg mouse brain slices (Fig. 4A). Finally, the m6A eraser, FTO, was detected in the nucleus and the soma of cells in the cortex, striatum, hippocampus, and cerebellum. FTO was also localised in the dendrites of neurons in the cortex and cerebellum in both Wt and aSyn Tg mouse brain slices (Fig. 4A). After analysis of the expression of these proteins calculated based on the fluorescence intensity, no significant differences were observed in the different regions of the brain when aSyn Tg mice were compared with Wt mice (Fig. 4B).

Our findings show that the m6A regulatory proteins – METTL3, YTHDF1 and FTO are present in the cortex, striatum, hippocampus, and the cerebellum in the mouse brain. In addition, we found that the expression levels of METTL3, YTHDF1 and FTO do not change in cortical brain tissue of mice.

### METTL3 is reduced in the aSyn Tg mouse post-synapse

**Fig. 5:**
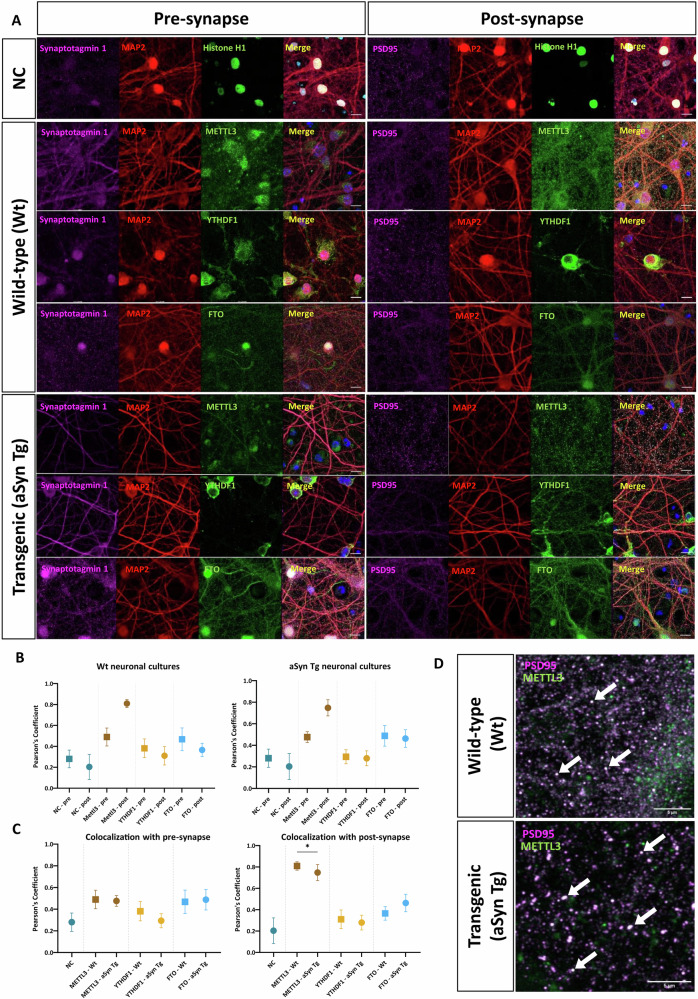
METTL3 is localised at the post-synapse in Wt and aSyn Tg DIV14 primary neurons. **A** Representative images of DIV14 primary neurons, fixed and stained with Histone H1, MAP2, PSD95, Synaptotagmin 1, METTL3, YTHDF1, and FTO in Wt and aSyn Tg neurons. Images are acquired using airy-scan at 63x magnification, scale bar = 10 µm. **B** Box and whisker plot representing the colocalisation of m6A regulators in Wt and aSyn Tg DIV14 primary neurons at the pre-and post-synapse. METTL3 colocalises with the post-synapse, (PCC, Wt = 0.810, aSyn Tg = 0.748). A significant difference (*, p-adj. value = 0.018) was observed between METTL3 at the post-synapse in Wt neurons when compared with aSyn Tg neurons. In the plots, the solid squares represent the pre-synaptic terminal and the solid circles represent post-synaptic terminal. **C** Box and whisker plot representing the colocalisation of m6A regulators at the pre-and post-synapse in Wt and aSyn Tg primary neurons. METTL3 colocalisation was significantly reduced in aSyn Tg neurons when compared with Wt at the post-synapse. Here, NC stands for negative control. In the plots, solid squares represent the Wt animals and solid circles represent the aSyn Tg animals. **D** Representative zoomed-in images of colocalization of METTL3 with post-synaptic maker, PSD95. The white arrows indicate the colocalisation of protein signal. Scale bar = 5 µm. Three replicate experiments were performed for Wt and aSyn Tg primary neuronal cultures and a minimum of 10–12 frames were acquired per experiment.

Based on our bioinformatics analysis, we observed that the majority of m6A genes were synaptic, so we asked if, and to what extent the m6A regulators are present at synapses acting as local regulators since the overall regulator levels remain unchanged. We used primary cortical neurons that allow higher imaging potential and performed co-staining of the three m6A regulatory proteins–METTL3, YTHDF1 and FTO alongside Syn1, pre-synaptic marker and PSD95, post-synaptic marker to study the localisation of these proteins at the synapse (Fig. 5A). Previous studies have indicated reduced synaptic activity in neurons derived from sporadic PD patients when compared with healthy controls^51,52^.

We (two independent experimenters) observed METTL3 was present within the nucleus as nuclear speckles and it significantly co-localised with PSD95, indicating its presence at the post-synapse in both Wt (PCC = 0.810 ± 0.039) and aSyn Tg (PCC = 0.748 ± 0.075) primary neurons (Fig. 5B, D). In contrast, METTL3 did not co-localise with Syn1 in Wt (PCC: 0.489 ± 0.086) or aSyn Tg (PCC: 0.476 ± 0.050) primary neurons, indicating no noticeable localisation at the pre-synapse (Fig. 5B). A significant downregulation of METTL3 was observed at the post synapse in aSyn Tg mice compared with Wt mice (*p* = 0.018) (Fig. 5C). Similarly, we investigated the compartmentalisation of YTHDF1, which did not overlap with either Syn1 (PCC: Wt = 0.382 ± 0.090, aSyn Tg = 0.294 ± 0.065) or PSD95 (PCC: Wt = 0.310 ± 0.088, aSyn Tg = 0.280 ± 0.069) (Fig. 5B). The m6A eraser protein, FTO, was identified within the cell nucleus and in some cases, in the soma of primary neurons. No significant co-localisation of FTO was observed at the pre- (PCC, Wt = 0.468 ± 0.109, aSyn Tg = 0.488 ± 0.095) or post-synapse (PCC, Wt = 0.366 ± 0.063, aSyn Tg = 0.463 ± 0.081) (Fig. 5B).

Overall, we observed that the expression of METTL3 was reduced at the post-synapse in aSyn Tg primary neurons suggesting a possible loss of METTL3 at the synapse in neurons with aSyn pathology.

### Primary neurons from aSyn Tg mice have a greater number of synapses than Wt animals

**Fig. 6:**
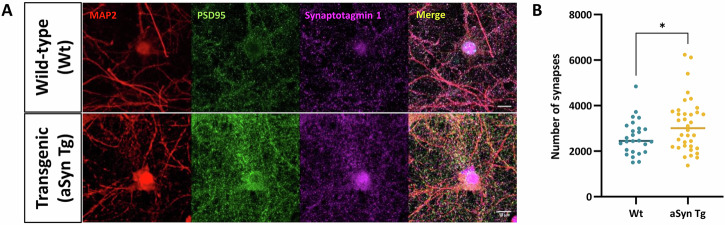
Increased number of synapses in aSyn Tg primary neurons when compared to Wt neurons. **A** Representative immunofluorescent images of DIV14 primary neurons stained with pre- (Syn1, magenta) and post-synaptic (PSD95, green) markers and MAP2 in red. Acquired at 63x magnification, airy scan mode. Scale bar = 10 µm. **B** Scatter plot for synapse counting for Wt and aSyn Tg mouse models with a significant reduction in the number of synapses (*, adj. p value = 0.044) in aSyn Tg primary neurons when compared with Wt. Three experiments were carried out for Wt and aSyn Tg neuronal cultures each and 10–12 frames per experiment were acquired for analysis.

Since we observed a reduction in the localisation of METTL3 at the synapse in aSyn Tg primary neurons, we wondered whether this has any influence on the synapse morphology and number, or vice versa. For this, we used primary cortical neurons and performed proximity analysis of the pre-and post-synaptic markers Syn1 and PSD95 respectively, using the IMARIS software (Imaris (RRID: SCR_007370)) and the data were analysed blindly to avoid bias (Fig. 6A). Our analysis revealed that the number of synapses in aSyn Tg primary neurons was significantly increased when compared with Wt neurons (Wt; mean = 2607 ± 763.1, aSyn Tg; mean = 3152 ± 1187, *p* = 0.044) (Fig. 6B).

## Discussion

The involvement of epitranscriptomics in PD is still not well understood. Previous studies have reported an increase in the levels of m6A at the 5’UTR in a drosophila model of Alzheimer’s disease, which suggests that increased m6A modification and altered activity of its associated proteins may play a role in the response of the brain to Aβ42^53^. However, since not much is known in the field of synucleinopathies, our objective in this study was to unravel the role of m6A RNA methylation in the A30P aSyn Tg mouse model of PD/synucleinopathy, in disease and physiological ageing.

Our findings revealed that 99.5% of the transcripts were differentially hypermethylated in 15 mo vs. 3 mo Wt mice suggesting that ageing might contribute to a significant increase in m6A methylation. This observation aligns with a recent study that has demonstrated an increase in m6A methylation during ageing in both mice and humans^35^. This indicates that the accumulation of m6A methylation marks might be a feature of ageing in the brain, potentially influencing various biological processes. It has also been shown that these epigenetic changes arise mainly in young and aged mice, while during the adolescent stage these genes are stabilised^54^. In contrast, when comparing 15 mo aSyn Tg mice with their 3 mo counterpart littermates, more transcripts were hypomethylated. This decrease in methylation with age in aSyn Tg mice might suggest that the loss of methylation might be related to aSyn expression or pathology, or a result of ageing. There is a possibility that the overexpressed aSyn is somehow interacting with the m6A regulators and inhibiting methylation. Previous studies have shown that the 6-hydroxydopamine rat PD model has significantly reduced m6A levels in the striatal brain region and in the cellular model of PD^55^. Similarly, a reduction in the total levels of m6A was also reported in the MPTP-treated PD mouse model and in MPP + -treated MN9D cells^56^.

The effect of aSyn expression was more directly studied in the comparison between 3 mo aSyn Tg and Wt mice. The increase in methylation suggests that aSyn expression may be linked to enhanced methylation activity in early life. However, in 15 mo aSyn Tg vs. Wt mice, we observed more hypomethylated genes, suggesting that A30P aSyn expression, and likely pathology, might interfere with the writing or erasing of methylation marks during ageing. Importantly, our results are in accordance with a recent publication suggesting that the levels of m6A are significantly lower in PD patients^46^.

Interestingly, based on GO term analysis, we found that differentially methylated transcripts were predominantly associated with synaptic function and that methylation was lost with ageing. Hypermethylation of transcripts involved in the synaptic processes might lead to an inhibition of mRNA translation and thereafter reduced functionality of the synapse^57,58^. As it has been observed that aSyn expression leads to hypomethylation during ageing, there is a possibility that this inhibition is alleviated and the translation of genes is recovered. Therefore, downregulation of methylation might retrieve synaptic function. This is particularly relevant as more than half of the causative genes and risk factors in PD have been identified to function at the synapse^59^ and this change in methylation pattern might indirectly affect the functioning of the synapse. Therefore, the increase in hypomethylated genes involved in synaptic processes suggests an increase in the proteins they encode for. Considering that different epigenetic mechanisms occur locally at the synapse; this underscores their potential importance in synaptic regulation^60^. Additionally, GO term analysis of the differentially expressed transcripts showed a strong enrichment in immune response-related pathways. Notably, m6A is implicated in the regulation of inflammation under physiological and pathological conditions, known as “inflammatory ageing”, or inflammaging. This is a chronic inflammatory state arising during ageing and it is characterised by an increase in pro-inflammatory factors such as tumour necrosis factor (TNF-α), interleukins (IL-1β and IL-6), and C-reactive protein (CRP), to name just a few^61^.

The assessment of total m6A methylation marks showed a significant reduction in the cortex of 12 mo aSyn Tg mice when compared with age-matched Wt mice. This suggests that the overall m6A methylation decreases in the ageing brain. This finding is consistent with recent studies that reported a predominant reduction in m6A methylation in the frontal cortex and cingulate cortex from PD patients^46,62^. In vitro studies have also reported a significant reduction in m6A levels in the cellular PD models^56^. Despite this, we observed similar expression levels of m6A regulatory proteins. However, our findings are consistent with those from a recent study reporting that the levels of YTHDF1 and FTO mRNA remain unchanged in PD. However, a reduction in the levels of METTL3 mRNA in PD patients was also reported in that study, in contrast to what we observed^46^. Some studies have also reported up-regulation of ALKBH5, IGF2BP2 in the substantia nigra while, YTHDF1 and fragile X mRNA (FMR1) were downregulated. In the striatum, an up-regulation of FMR1 and Cbl proto-oncogene like 1 (CBLL1) and down-regulation of METTL3, RBM15 and IGF2BP3 was observed^42^.

Although we found no significant differences in the levels of m6A regulatory proteins, we observed that these three proteins were present in the cortex, striatum, hippocampus, and cerebellum. Our findings are consistent with those from previous studies reporting that the m6A-methyltransferase complex, including METTL3, is predominantly found in the nucleus, but that it can also be localised in the cytoplasm^15,25,63^. Methylation generally occurs co-transcriptionally in the nucleus, while reader proteins occur largely at the cytosol, including YTHDF1^64^. FTO, on the other hand, is localised both in the nucleus and in the cytosol^65^. These observations suggest that an inhibitory factor affecting their function and aSyn aggregation is potentially playing a role. Previous studies have shown that aSyn binds to histone proteins, which may alter the protein surface due to post-translation modifications of histone tails, altering their affinity for DNA and other histones^66^. Another study showed that aSyn expression increases histone-H3 lysine-9 (H3K9) methylation, suggesting a possible interaction of aSyn with epigenetic writer proteins^67^. This could constitute a mechanism through which aSyn affects gene methylation, ultimately affecting synaptic functioning^68,69^.

Given that most of the hypermethylated genes were synaptic, we assessed the presence of these regulators at the synapse. Our findings suggest the presence of METTL3 at the post-synapse, indicating that the enzyme might be locally active at the synapse. The dynamic nature of m6A methylation may be localised to different regions within a cell, depending on the temporal requirements of the respective genes^70,71^. In another study, m6A regulatory proteins were found localised adjacent to the synapse and in the dendrites, also suggesting the sub-cellular localisation of m6A methylated mRNA which might lead to synaptic modifications^72,73^. Our findings revealed that the expression of METTL3 is reduced at the post-synapse in aSyn Tg DIV14 neurons, suggesting a possible loss of METTL3 at the post-synapse. Due to this reduction at an early stage, the nucleus might be sending hypermethylated transcripts to the dendrites to compensate for this loss. However, this may be lost during ageing. Based on our bioinformatics analysis, we observed that methylated transcripts associated with synaptic function were reduced in aSyn Tg mice with ageing. This suggests that a reduction in the m6A methylation in synaptic genes may be directly linked with the reduced METTL3 localisation at the synapse in aSyn Tg neurons. Additionally, our findings indicate a significant increase in synapse number in aSyn Tg mice, which suggests that this might be a compensatory mechanism to overcome the reduction in localisation of METTL3 at the synapse in aSyn Tg mice. We acknowledge some limitations of this study. For the m6A RNA sequencing, only male mice were used for consistency with our previous work. In addition, including female animals, would require assessing their oestrous cycle, and since we had a limited number of progenies, it was not feasible to include them in our study.

In conclusion, our study highlights the critical role of m6A RNA methylation in the progression of PD and its implications in ageing, particularly focusing on the A30P aSyn mouse model. Our findings indicate that aSyn expression/pathology may influence RNA methylation patterns, leading to reduced levels of m6A in mice. The differentially methylated transcripts were predominantly synaptic, indicating that m6A modifications play a crucial role in synapse physiology and potentially contribute to the synaptic dysfunction observed in PD^74^. Although we observed no significant changes in the levels of m6A regulatory proteins, the reduction in METTL3 at the synapse in aSyn Tg mice informs on the importance of subcellular compartmentalisation of these proteins in the regulation of RNA methylation. Therefore, our research provides new insights into the epigenetic mechanisms underlying PD and underscores the need for further exploration of m6A RNA methylation as a potential therapeutic target for PD and other related synucleinopathies.

## Methods

### Resource availability

#### Lead contact for reagent and resource sharing

Further information and requests for resources and reagents should be directed to and will be fulfilled by the lead contact, Tiago F. Outeiro (tiago.outeiro@med.uni-goettingen.de).

### Experimental model and subject details

#### Animals

Five male homozygous [A30P] aSyn transgenic (aSyn Tg) mice (C57BL/6J-Tg(Th-SNCA*A30P*A53T) 39Eric/J under the Thy-1 promotor, Strain #:008239, RRID: IMSR_JAX:008239) and five wild type (Wt) (C57BL/6 J) littermates, 3 mo and 15 mo were included in this study for RNA sequencing and analysis, protein estimation, and imaging. Additional animals used for each experiment are mentioned in the respective section. Animals were housed in standard cages with 12 h light/12 h dark cycle and *ad libitum* access to food and water in the animal facility of the University Medical Centre Göttingen (Göttingen, Germany). Animal procedures were performed in accordance with the European Community (Directive 2010/63/EU); and institutional and national guidelines under the Lower Saxony State Office for Consumer Protection and Food Safety (LAVES) (license number 19.3213). Animals were anesthetised with carbon dioxide (CO_2_) and then sacrificed using cervical dislocation. For tissue processing, the brain was surgically extracted from the skull and was further dissected to isolate the cortex and midbrain. The tissue was snap-frozen in liquid nitrogen and preserved at −80 °C until use.

For the preparation of brain slices, the animals were perfused first with 1x phosphate buffer saline (PBS), followed by 4% paraformaldehyde (PFA). The whole brain was dissected out of the skull and placed overnight in 4% PFA at 4 °C. The following day, the brain was washed once with 1x PBS and then placed in 15% saccharose, and lastly in 30% saccharose with 0.1% sodium azide (NaN_3_). The brains were placed at 4 °C until use. Sagittal cryo-sections of 30 µm thickness were sectioned using a cryostat (Leica CM3050 S) for each mouse brain. The sections were placed in 1x PBS with NaN_3_ until they were used for immunohistochemistry (IHC) protocol.

### RNA extraction

For brain tissue, TRIzol reagent (Invitrogen, CA, USA) and ceramic beads (1.4 mm diameter ceramic beads, 91-PCS-CK14B, PEQLAB Biotechnologie, Germany) were added in vials with frozen tissue and homogenisation was performed with the Precellys 24 tissue homogeniser (Bertin Instruments) at 65 000 g for 2 × 30 s runs with a 30 s break in between. RNA isolation was performed with TRIzol reagent according to the manufacturer’s instructions. Briefly, 0.5 ml of TRIzol was added to the tissue samples and incubated for 5 min before homogenisation. After homogenisation, 0.1 ml of chloroform was added to each tube. The tubes were shaken vigorously and incubated at RT for 2–3 min. The tubes were centrifuged at 14,000 *g*, for 10 min at 4 °C. The top aqueous layer was carefully transferred to a fresh tube. Total RNA was then precipitated using Clean and Concentrator 5, Zymo research kit (Zymo Research, #R1014). Following RNA extraction and precipitation, RNA was dissolved in RNase-free water and concentration and quality were estimated with NanoDrop 1000 spectrophotometer (Thermo Fisher Scientific, MA, USA) by measuring absorption at 260 nm and the ratios 260/280 nm and 230/280 nm respectively.

### MeRiP

Total RNA was treated with DNase to remove any DNA remnants and further, 5–10 µg of total RNA was depleted for rRNA using the RiboMinus Eukaryote Ribosomal Removal Kit (Invitrogen). The large RNA fraction (>200 bp) was fragmented to a fragment size of approximately 100–120 nt by RNA Fragmentation Reagent at 70 °C for 5 min (#AM8740, Thermo Fisher Scientific, Waltham, MA). 5% of this RNA sample was kept as inputs and the rest was subjected to immunoprecipitation (IP). 300 ng of fragmented RNA was used for each IP. 3 µg of anti-m6A antibody was incubated with the RNA IP buffer (0.2 M Tris-HCl pH 7.5, 0.5 M NaCl and 2% (vol/vol) Igepal CA-630) for 2 h at 4 °C with constant rotation. Antibody-RNA conjugates were incubated with 30 μl of Protein A/G beads overnight (ON) at 4 ˚°C with rotation in 500 μl IP buffer supplemented with 200 units. The beads with immunoprecipitated RNA were washed with IP buffer five times, and further washed with low-salt (50 mM Tris pH 7.4, 50 mM NaCl, 1 mM EDTA, 1, 0.1% NP-40, 0.1% SDS) and high-salt (same as low-salt but with 500 mM NaCl) buffers at 4 °C with rotation to remove any nonspecific binding. RNA was eluted by elution buffer with 6.7 mM m6A (in IP buffer). Eluted RNA was cleaned before proceeding to library preparation.

### Library preparation and sequencing

Samples were prepared for sequencing using the SMARTer Stranded Total RNA-Seq Kit v2 — Pico Input Mammalian (Takara) according to the manufacturer’s protocol. All the RNA obtained from the IP samples was used for library preparation, for input samples, 2 ng was used. The libraries were amplified for a total of 12 cycles for input and 16 cycles for IP samples. RNA-Seq samples were prepared with the TruSeq Stranded mRNA Library Prep Kit (Illumina) according to the manufacturer’s instructions for which, 50 ng of rRNA-depleted RNA from each of the inputs was used. Prepared libraries were sequenced in a Hiseq 2000 System (Illumina) for 50 cycles in single-end reads. Additional metadata is available via the GEO database (GSE275432).

### Bioinformatic analysis of MeRIP-seq and RNA-seq

Raw reads were processed and demultiplexed using bcl2fastq (v2.20.2), and low-quality reads were filtered out with Cutadapt v1.11.0^75^. Filtered reads were mapped to the mouse (mm10) genome using the STAR aligner v2.5.2b^76^. The resulting bam files were sorted and indexed, and the unmapped reads were removed using SAMtools v1.9.0^77^. Methylation sites were determined using MeTPeak v1.0.0^78^, and differential methylation (hypo-and hyper-methylated regions) was assessed with ExomePeak v2.16.0^79^ using 3 mo samples as control and 15 mo as a treatment for the first comparison and the second comparison, Wt samples were used as controls while aSyn Tg was used as treatment. An adjusted p-value (p-adj, also termed as FDR [false discovery rate]) cutoff of 0.05 and FC cutoff of 1.2 or 1.5 were used as indicated in the text. Only consistently significantly, differentially methylated peaks were used, unless indicated.

For RNA-seq analyses, read counts were obtained with subread’s featureCounts v1.5.1^80^ from the bam files of input samples. Differential gene expression was determined by DESeq2 v3.5.12^81^ using normalised read counts and correcting for covariates detected by RUVseq v1.16.1^82^. Cutoffs of p-adj ≤ 0.05, FC ≥ 1.2, and BaseMean ≥ 50 were applied to the results (Supplementary Fig. S1A, B). Background expressed genes were determined for each region as those genes with a BaseMean > 50 in the corresponding input sample.

Methylation sites were determined using MeTPeak 1.0.0^78^ and differential methylation was assessed with exomePeak 2.16.0^79^. To differentiate between hypo- and hypermethylated genes, an adjusted p-value (p-adj, also termed as FDR [false discovery rate]) cutoff of 0.05 and FC cutoff of 1.2 or 1.5 were used and only the consistently significantly differentially methylated peaks were used.

### GO analysis

GO term enrichment analyses were performed using the App ClueGO v2.5.3^83^ in Cytoscape 3.7.2^84^, with GO Term Fusion enabled to collapse terms containing very similar gene lists and using a custom background corresponding to expressed genes in the corresponding species as obtained from RNA-seq results of the corresponding input samples of the MeRIP experiments. GO term tables for biological processes, cellular components, pathways, and KEGG were produced and are labelled accordingly in the figures. The resulting enriched GO terms were visualised with a custom script using ggplot2 v3.3.5^85^ playing the adjusted p-value (p-adj) for the GO term, the number of genes from the list that belong to said term, and the percentage of the total genes in the GO term that are present in the list. Synaptic GO enrichment analyses were performed with SynGO (v1.1, syngoportal.org)^50^.

### Additional bioinformatics packages and tools

Scripts and analysis pipelines were written in R (3.5.2)^86^. Peak annotation was performed with Annotatr v1.8.0^87^. Guitar plots were produced with the Guitar v1.20.1^88^ R package. Volcano plots were generated with plot.ly/orca v4.9.4.1^89^. Area-proportional Venn diagrams were produced with BioVenn (www.biovenn.nl), and multiple list comparisons performed with Intervene/UpSet (asntech.shinyapps.io/intervene/). Mouse/human homologues were determined by their annotation in NCBI’s HomoloGene database using the HomoloGene (v1.4.68.19.3.27) R package. Odds ratios and p values to determine significance in overlapped datasets were calculated with the GeneOverlap R package v1.18.0^90^. Microscopy images were pre-processed with Fiji, and quantification was automated in Cell Profiler (cellprofiler.org)^91^. Graphs, heat maps, and statistical analyses were performed on GraphPad Prism version 9.3.1 for Mac.

### Neuronal cultures

Primary cortical neuronal cultures were prepared from both aSyn Tg and Wt mice as previously described^92^. Cells were seeded on coverslips coated with poly-L-ornithine (0.1 mg/mL in borate buffer; PLO) (Sigma-Aldrich, MO, USA) or culture plates (Corning, Merck, Darmstadt, Germany) for immunocytochemistry and maintained in neuronal cell culture medium (Neurobasal medium, Gibco Invitrogen, CA, USA), supplemented with 1% penicillin-streptomycin (PAN Biotech, Aidenbach, Germany), 0.25% GlutaMAX (Gibco Invitrogen, CA, USA), and 2% B27 (B-27™ Plus Supplement (50X), Gibco, #A3582801). The cells were maintained at 37 °C with 5% CO_2_, and one-third of the medium was replaced with fresh media every 3–4 days to replenish the nutrients.

### Immunocytochemistry

Primary neurons were washed with 1x PBS (PAN Biotech, Aidenbach, Germany) and fixed with 4% of PFA for 20 min at room temperature (RT). The cells were permeabilized with 0.1% Triton X-100 (Sigma-Aldrich, MO, USA) for 10 min and then blocked with 1.5% normal goat serum (NGS) in 1x PBS (Sigma-Aldrich, MO, USA) for 1 h at RT. The cells were then incubated with the primary antibodies diluted in blocking solution (FTO (mouse, Abcam – ab92821, 1:1000), METTL3 (rabbit, Cell Signalling - #96391S^93^, 1:1000, RRID: AB_2800261), YTHDF1 (rabbit, Proteintech 17479-1-AP, 1:1000), MAP2 (rabbit, Proteintech – #17490-1-AP, 1:1000), postsynaptic density protein 95 (PSD95) (mouse, Synaptic systems 124011, 1:1000), PSD95(D74D3) (rabbit, Cell Signalling - #3409, 1:1000), synaptotagmin 1 (Syn1) (mouse, Synaptic systems - #105311, 1:1000) synaptotagmin1 (rabbit, Synaptic systems – 105102/3, 1:1000)) overnight at 4 °C, with constant shaking at a speed of 40–50 rpm. The following day, cells were washed three times for 10 min each with 1x PBS (PAN Biotech, Aidenbach, Germany) and then incubated with fluorescently conjugated secondary antibodies (Alexa Fluor 488 donkey anti-mouse IgG – Invitrogen, Alexa Fluor 488 donkey anti-rabbit IgG - Invitrogen, Alexa Fluor 555 donkey anti-mouse IgG – Invitrogen, Alexa Fluor 555 donkey anti-rabbit IgG – Invitrogen, Alexa fluor 633 goat anti-chicken IgG – Invitrogen, all with a dilution of 1:1000) for 2 h at RT, on a shaker at 40–50 rpm. Lastly, nuclei were counter-stained with DAPI (Carl Roth, Karlsruhe, Germany) and mounted on glass slides (Epredia™ SuperFrostPlus™ Adhesion) with mounting media (Invitrogen™ Fluoromount-G™) for microscopy.

### Immunohistochemistry

The free-floating tissue was carefully transferred from PBS and NaNH_3_ to 1x tris-buffered saline (TBS) in a 24-well plate with immunohistochemistry (IHC) grids. The tissue was washed three times for 5 min each with 1x TBS to remove the remnants of NaN_3_. Following this, tissue antigen retrieval was performed using citrate buffer (10 mM citrate, 0.05% Tween 20, pH 6.0) at 90 °C for 30 min in a water bath. The tissue samples were allowed to cool down for 20 min or until they reached RT. Tissue sections were washed thrice for 10 min each with 1x TBS and then incubated with 0.3% Triton X-100 for 20 min. Without washing, the tissue slices were incubated directly in a blocking solution (5% BSA, and 1% NGS in 1x TBS) for 2 h at RT. The tissue sections were then incubated for 48 h with antibodies diluted to1:200 in blocking solution (FTO (mouse, Abcam – ab92821, 1:1000), METTL3 (rabbit, Cell Signalling - #96391S, 1:1000), YTHDF1 (rabbit, Proteintech #17479-1-AP, 1:1000), MAP2 (rabbit, Proteintech – #17490-1-AP, 1:1000)) at 4 °C, on a shaker. The following day, tissue sections were washed thrice for 10 min each with 1x TBS at RT. Afterwards, the tissue sections were incubated in secondary antibody at a dilution of 1:400 (Alexa Fluor 488 donkey anti-mouse IgG – Invitrogen, Alexa Fluor 488 donkey anti-rabbit IgG - Invitrogen, Alexa Fluor 555 donkey anti-mouse IgG – Invitrogen, Alexa Fluor 555 donkey anti-rabbit IgG – Invitrogen, Alexa fluor 633 goat anti-chicken IgG - Invitrogen) overnight at 4 °C on a shaker. The following day, the tissue was washed thrice for 10 min each with 1x TBS to remove the unbound secondary antibody. The tissue sections were incubated for 10 min in DAPI (Carl Roth, Karlsruhe, Germany) followed by one 10 min wash with 1x TBS. The tissue sections were then mounted onto glass slides (Epredia™ SuperFrostPlus™ Adhesion) and left to dry completely away from the light, at RT. 8–10 µl of mounting media (Invitrogen™ Fluoromount - G™) was applied as a drop onto each section on the glass slide and a glass coverslip (Avantor, 631-0147, 0.16-0.19 mm thickness, #1.5) was carefully placed on the tissue avoiding air bubbles.

### Western blots

Cortical brain tissue was extracted from three adult mice per age group (age group 2 mo, 6 mo and 12 mo) from each Wt and aSyn Tg mice. The tissue from adult mice was lysed using homogenisation beads (1.4 mm diameter ceramic beads, 91-PCS-CK14B, PEQLAB Biotechnologie, Germany) in RIPA buffer (50 mM Tris, pH 8.0, 0.15 M NaCl, 0.1% SDS, 1.0% NP-40, 0.5% Na-Deoxycholate, 2 mM EDTA) supplemented with protease and phosphatase inhibitors cocktail (cOmplete™, Mini Protease Inhibitor Cocktail, Roche). The homogenisation was performed in Precellys 24 tissue homogeniser (Bertin Instruments) at 65,000 × *g* for 2 × 30 s runs with a 30 s break in between. Protein concentrations in the lysates were determined by the BCA protein assay (Pierce Biotechnology, #23225) following the manufacturer’s protocol. The spectrophotometric recordings were performed using a plate reader (Tecan, Infinite® M200 pro) at 562 nm. Volume required for 20 μg of protein per sample was calculated and Laemmli sample buffer (250 mM Tris-HCl pH 6.8, 10% SDS, 1.25% Bromophenol Blue, 5% ß-mercaptoethanol, 50% glycerol) was added accordingly (1:5 dilution of Laemmli: protein). The proteins were denatured for 5 min at 95 °C, with 600 rpm in a thermal shaker. The samples were loaded on a 12% SDS-PAGE electrophoresis gel and separated at 120 V for approximately 1 h. The proteins were then transferred to PVDF membranes (iBlot2 Transfer Stack, PVDF, Life, #IB24002X3) using iBlot2 (Invitrogen, CA, USA) using the P0 protocol for 7 min. Membranes were blocked with 5% BSA (Sigma Aldrich, #AG9418) in TBS (pH 8) with 0.05% Tween-20 (TBS-T) and then incubated separately with the primary antibodies for FTO (mouse, Abcam – ab92821, 1:5000), METTL3 (rabbit, Cell Signalling - #96391S, 1:1000), YTHDF1 (rabbit, Proteintech #17479-1-AP, 1:2500) overnight in 5% BSA (Sigma-Aldrich, MO, USA), and positive control GAPDH (GAPDH (14C10) rabbit, Cell Signalling, #2118, 1:10000) in TBS-T at 4 °C. After three washes with TBS-T, membranes were incubated for 2 h with horseradish peroxidase (HRP) conjugated secondary antibodies (Cytiva sheep anti-mouse IgG, 1:8000 dilution, Cytiva donkey anti-rabbit IgG, 1:8000 dilution). Following incubation, membranes were washed three times with TBS-T for 10 min each and developed in a chemiluminescence system (Fusion FX Vilber Lourmat, Vilber, France) using chemiluminescent HRP substrate (Millipore, MA, USA). Intensities of specific bands were normalised to a protein loading control.

### m6A fluorescence ELISA

m6A RNA methylation ELISA (m6A RNA methylation, Abcam, #ab233491) was used for the detection of m6A RNA methylation in the cortical brain and midbrain tissue from young, 3 mo and aged, 12 mo/15 mo Wt and aSyn Tg mice. For this experiment, we used cortical mouse brain tissue from Wt mice each at 3 mo and 12 mo (six 3 mo and six 12 mo), and aSyn Tg mice (six 3 mo and five 12 mo). Midbrain tissue was used from Wt mice (three 3 mo and four 15 mo) and aSyn Tg mice (five 3 mo and four 15 mo). This protocol ensures that the total m6A methylation is measured in both mRNAs and other ncRNAs (tRNA, rRNA and snRNA). The protocol was performed according to the manufacturer’s protocol. Briefly, total RNA isolated from the tissue was added to bind to the assay wells. The wells were then washed to remove the unbound RNA and diluted capture antibody was added to the wells. Unbound capture antibody was washed out with multiple washing steps. Diluted detector antibody was then added along with fluoro-enhancer. The wells were washed again and incubated with fluoro developer mixture to initiate the fluorescence reaction. The fluorescence was then measured at 590 nm using plate reader (Tecan, Infinite® M200 pro).

### Microscopy

Images of the immunofluorescence-stained neurons for three experiments in total, and capturing at least 10 regions of interest per experiment, and brain tissue were acquired using a confocal microscope ZEISS LSM900. The images for the neuronal cell culture were acquired as a Z-stack using 63x magnification with Airyscan mode. Brain slices were imaged at 40x magnification, with tiles in confocal mode of acquisition.

## Statistical analysis

The cell culture images for m6A regulators and their colocalization at the synapse were analysed using FIJI with the plugin JaCoP, while the synapse counting was performed using IMARIS. IMARIS analysis was performed following the protocol from the Queensland Brain Institute. The protein signals were further classified inside the dendritic or outside using membranes based on the MAP2 signals. The signals were then divided into four groups, representing PSD95 inside the neuron, PSD95 outside the neuron, Synaptotagmin1 (Syn1) inside the neuron and Syn1 outside the neuron. When the distance of the signal for PSD95 inside the neuron was at <500 nm from the signal for Syn1, it was considered a synapse. Colocalisation of synaptic markers with m6A regulators was estimated using Pearson’s correlation coefficient (PCC) estimated using the JaCoP plugin in ImageJ, FIJI. All IHC images were also analysed for their expression levels using FIJI software. Data analysis was performed using the GraphPad for Windows (GraphPad Software version 9, La Jolla California USA, https://www.graphpad.com) and JMP statistical analysis software. For group comparisons, one-way ANOVA with Tukey-Kramer’s test was used, while comparisons of two groups of means were done with an unpaired student’s t-test. All data are expressed as mean ± SD. Differences are considered significant with *p* < 0.05 (*p < 0.05, **<0.01, ***<0.001).

## Supplementary information

Supplementary Data.

## Data Availability

The datasets used and/or analysed during the current study are available from the corresponding author upon reasonable request. All raw data files for MeRIP-seq are available on the GEO database (GSE275432). To review GEO accession GSE275432 on https://www.ncbi.nlm.nih.gov/geo/query/acc.cgi?acc=GSE275432, enter token otebmkqmxdkrlot into the box.
