# Supplementary material for: The epitranscriptomic m6A RNA modification modulates the synapse in ageing and in a mouse model of synucleinopathy
Source: NPJ Parkinsons Dis. 2026 May 6;12:117. doi: 10.1038/s41531-026-01362-3 (PMC13180986; doi:10.1038/s41531-026-01362-3)
Supplement: Supplementary file 1 — Supplementary Data. [file 41531_2026_1362_MOESM1_ESM.pdf]

Supplementary Data

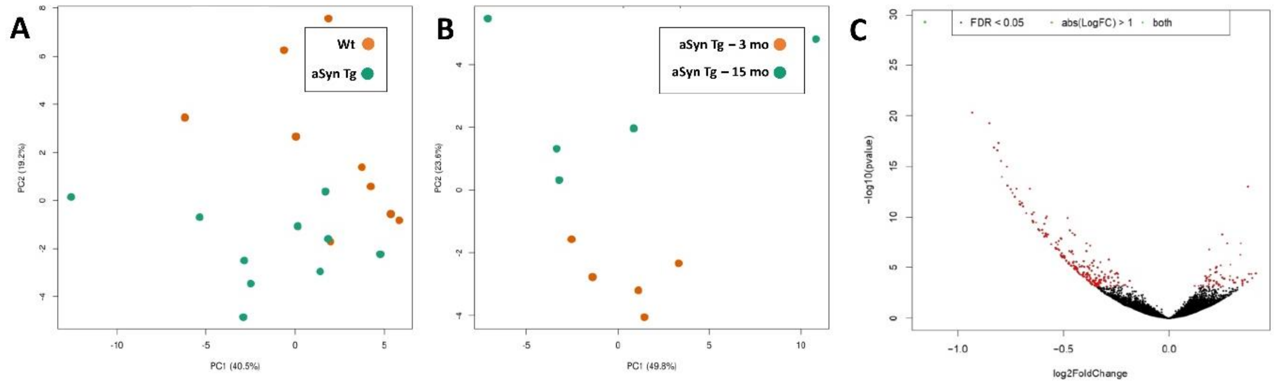

Supplementary Figure S1. Gene expression analysis in aSyn Tg and Wt mice. (A) Principal component analysis (PCA) plot of total Wt vs. aSyn Tg mice (PC1 = 40.5%, PC2 = 19.2%). (B) PCA plot of aSyn Tg – 3 mo and 15 mo mice, showing a clear segregation (PC1 = 49.8%, PC2 = 23.6%). (C) Volcano plot showing that the expression of most of the genes is down regulated in aSyn Tg mice, due to ageing (FDR < 0.05, abs(logFC) >1), aSyn Tg 15 mo vs. 3mo.

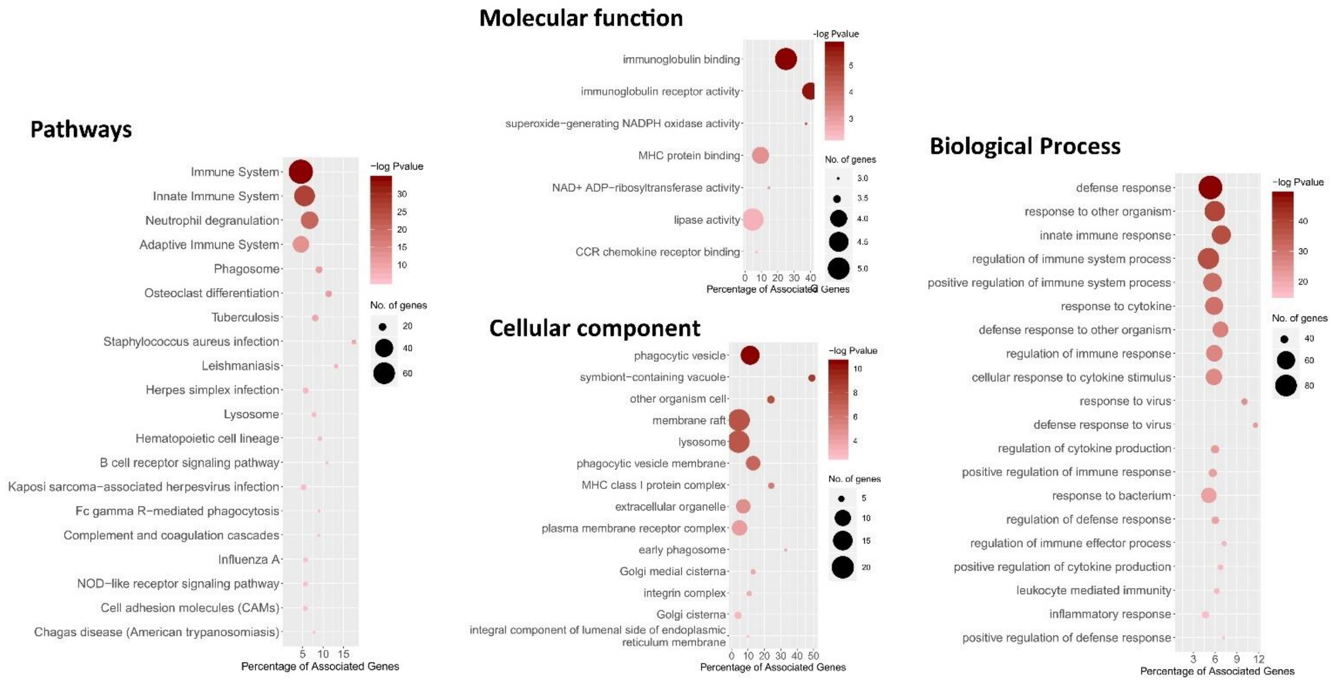

Supplementary Figure S2. Differentially expressed gene pathways in aSyn Tg 15 mo vs. 3 mo. GO term analysis comparing the enrichment of pathways, molecular function, cellular components, and biological processes of differentially methylated genes. Most pathway enrichment was observed in immune system and immune response linked pathways including – immunoglobulin binding, phagocytic vesicle, defense response and innate and adaptive immune response, FC > 1.2, FDR = 1.5.

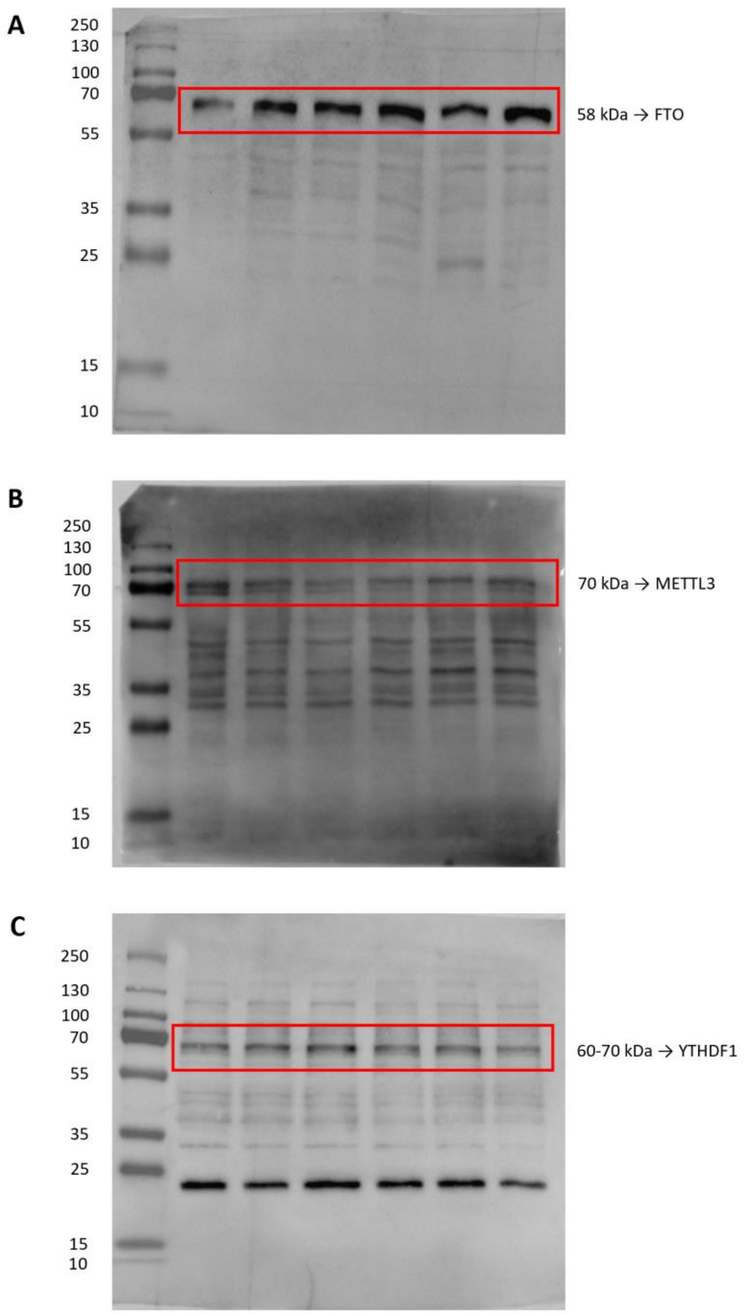

Supplementary Figure S3. Raw Western blot membranes for the assessment of m6A regulators FTO (A), METTL3 (B), and YTHDF1 (C) from Wt and aSyn Tg mouse brain tissue. These results have been elaborated in figure 3 in the main text.

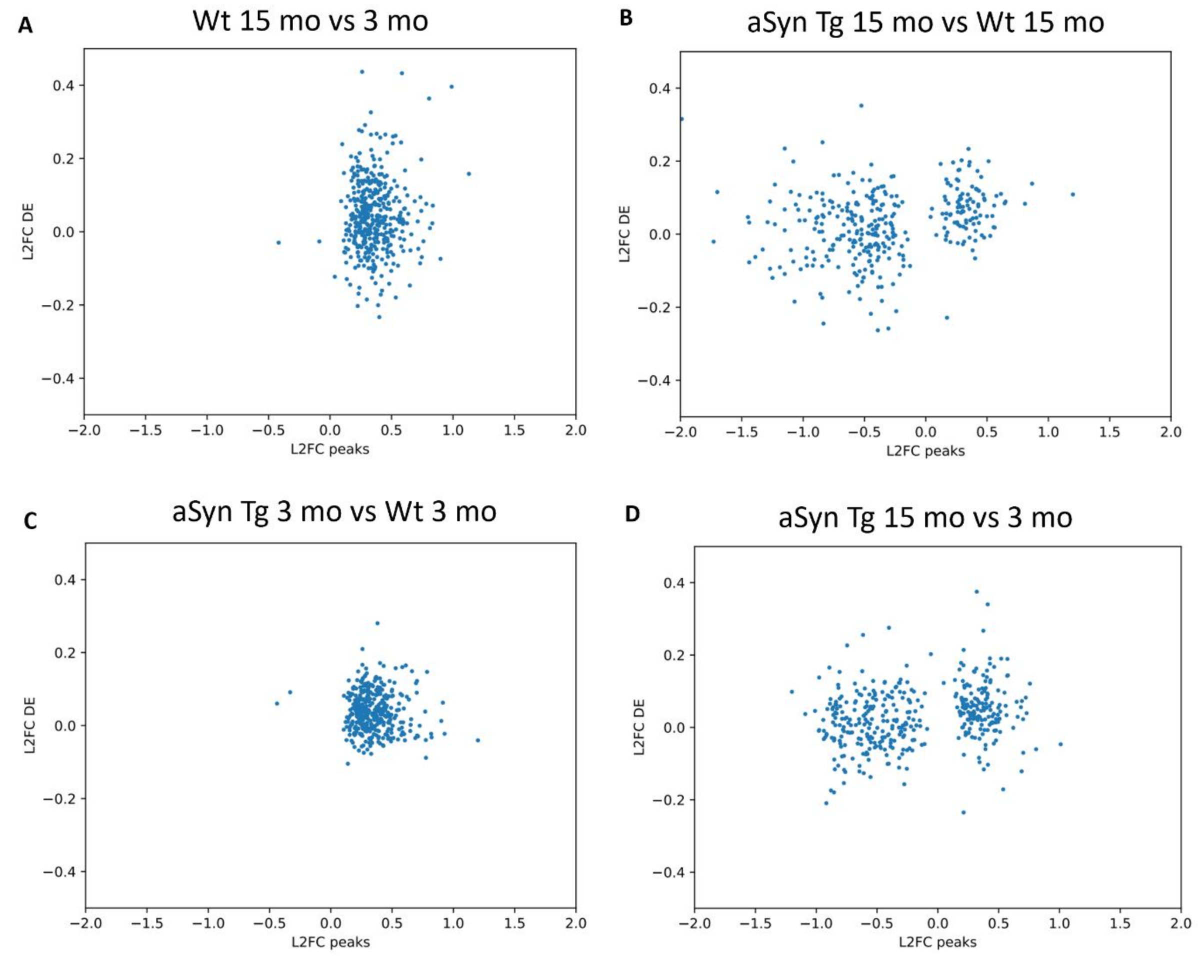

Supplementary Figure S4. No correlation was observed between the m6A peaks and mRNA levels when compared in (A) Wt 15mo vs 3 mo, (B) aSyn Tg 15 mo vs Wt 15mo, (C) aSyn Tg 3mo vs Wt 3mo, and (D) aSyn Tg 15 mo vs 3 mo.
